# Temporary employment and tooth loss: a cross-sectional study from the J-SHINE study

**DOI:** 10.1186/s12903-018-0488-4

**Published:** 2018-02-21

**Authors:** Yukihiro Sato, Toru Tsuboya, Richard G. Watt, Jun Aida, Ken Osaka

**Affiliations:** 1 Department of International and Community Oral Health, Tohoku University Graduate School of Dentistry, 4-1, Seiryo-machi, Aoba-ku, Sendai, Miyagi Japan; 2 Department of Epidemiology and Public Health, University College London, Gower Street, London, WC1E 6BT UK

**Keywords:** Employment status, Number of teeth, Non-regular employment

## Abstract

**Background:**

Temporary employment leads to psychological distress and higher mortality, but data on its associations with oral health is limited. We examined whether having the experience of temporary employment was associated with tooth loss among working adults in Japan.

**Methods:**

We conducted a cross-sectional study from the 2010–2011 Japanese Study on Stratification, Health, Income, and Neighborhood study that analyzed 2652 participants aged 25–50 years (men = 1394; women = 1258). Independent variable was changes in employment status (continuous regular employment and the experience of temporary employment). Dependent variable was self-reported tooth loss (none, 1 tooth, 2 teeth, 3 teeth, 4 teeth, and more than 4 teeth). Covariates were sex, age, years of education, self-rated household economic status in early life at 5 years old, marital status, number of family members in the household, history of diabetes, and body mass index. We conducted a negative binomial regression analysis to estimate prevalence rate ratios (PRRs) and 95% confidence intervals (95%CIs) for tooth loss. We also confirmed the interaction term between changes in employment status and sex.

**Results:**

The median age of the participants was 37 years. The percentages of men and women who experienced temporary employment were 14.5% and 61.3%, respectively. Compared with continuous regular employment, the experience of temporary employment was significantly associated with tooth loss in both sexes after adjusting for the covariates (men: PRR = 1.50 [95%CI = 1.13, 2.00]; women: PRR = 1.42 [95%CI = 1.14, 1.76]). The interaction term between employment status and sex was not significant (*p* = 0.71).

**Conclusions:**

Temporary employment is adversely associated with oral health.

## Background

Oral diseases remain a significant public health problem due to their very high prevalence, major impact on quality of life [1], and costs on health care systems [2]. In addition, oral diseases are socially patterned and closely related to social deprivation [3]. Consequently, stark social inequalities in oral health are now a major public health concern [4].

Temporary employment has attracted the attention of health researchers in recent years, because it has significant adverse effects on health [5–9]. Owing to considerable changes in the labour markets, inferior working conditions such as temporary contracts and an imbalanced working organization have emerged as a significant risk factor for poor health [10]. Unstable employment, such as temporary contracts, has been regarded as being harmful to health [5], and therefore, employment status might worsen health inequalities through employment status [5]. Temporary employment also may be harmful to oral health because work stress might lead to smoking tobacco [11] and decreasing salivary flow, which increases the risk of periodontal disease [12]. In addition, temporary employees might experience more severe tooth loss than regular ones, because their incomes are in general lower than ones of regular employees and they often do not receive adequate social benefits, such as health pensions [13].

A few studies have examined the relationship between employment status and oral health, including some that examined the association between unemployment and oral health [14–16]. To our knowledge, only one cross-sectional study has reported significant associations between the workplace-related factors such as precarious employment status and poor self-rated oral health [17]. Our main hypothesis was that changes in employment status between regular and temporary employment would have a negative impact on tooth loss. The aim of this study was to examine whether the experience of temporary employment is associated with tooth loss among working adults in Japan.

## Methods

### Data sources and participants

**Fig. 1:**
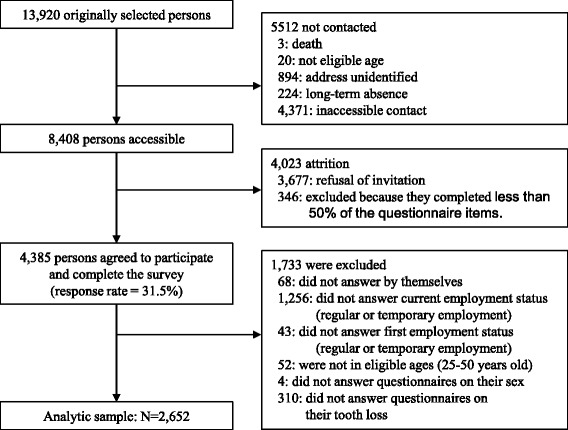
A flowchart of participants in the present study

We used data from the Japanese Study on Stratification, Health, Income, and Neighborhood (J-SHINE), which has been described in detail elsewhere [18]. This survey was conducted between July 2010 and February 2011. Target participants were adults aged 25–50 years old from 4 municipalities in Japan (2 in the Tokyo metropolitan area and 2 in neighboring prefectures). Figure 1 shows a detailed flowchart of participant selection. A total of 13,920 participants were probabilistically selected from the residential registry. Trained survey staff successfully contacted 8408 community dwelling adults, and 4385 participants agreed to participate in the survey (response rate 31.5%). The inclusion criteria were being 25–50 years of age and being regular or temporary employees at initial (previous) and current employment. The exclusion criteria were having missing values among the independent or dependent variables and not having answered the survey questions by themselves. We excluded 68 participants who did not answer the survey questions by themselves, 1256 participants who did not answer the question about current employment status (regular and temporary), 43 participants who did not answer the question about initial employment status (regular and temporary), 52 participants who were not aged 25–50 years old, 4 participants who did not indicate their sex, and 310 participants who did not answer the question about tooth loss. The analytic population was 2652 participants (the details are shown in Fig. 1).

### Study design

This study was a cross-sectional study.

### Independent variable: Changes in employment status

We obtained information about current employment status from the question, “What is your employment? If you have several jobs, please answer about your main job.” Respondents chose one answer from the following: “A president or an executive officer,” “Regular employment,” “Temporary employment,” “Contract employment,” “Part-time employment,” “Self-employed,” “Housekeeper,” “Subsidiary jobs,” and “Unknown.” We categorized participants who chose the answer regular employment into the regular employment group and participants who chose the answers temporary employment, contract employment, or part-time employment as temporary employment. We excluded those who chose president or executive officer, self-employed, housekeeper, subsidiary jobs, or unknown in the categorization of initial or current employment status (see Fig. 1).

We asked all participants whether they had changed jobs. Among only those who had changed jobs, we obtained information about their previous (initial) employment status using the same questions posed for current employment status. For the main analysis, we used the replies about current and initial employment status to prepare two categories for the independent variable: continuous regular employment and the experience of temporary employment. For a more analysis, we created four categories: continuous regular employment (regular employee at both times), regular to temporary employment (regular employee at initial employment and temporary employee currently), temporary to regular employment (temporary employee at initial employment and regular employee currently), and continuous temporary employment (temporary employee at both times).

### Dependent variable: Self-reported tooth loss

Dependent variable was self-reported tooth loss. We obtained this information using the question, “How many teeth have you had removed/extracted (excepting tooth extraction for orthodontic treatment, wisdom tooth extraction, and primary teeth)?” Respondents chose one of the following: “None” (scored 0), “1 tooth” (scored 1), “2 teeth” (scored 2), “3 teeth” (scored 3), “4 teeth” (scored 4), and “more than 4 teeth” (scored 5). We used self-reported tooth loss as a count variable.

### Covariates

We regarded the following factors as potential confounders, and included them in the multivariable adjusted models: age (categorized as 25–30, 30–35, 35–40, 40–45, or 45–50 years) and sex (men or women). Health status variables that may be related to employment status and tooth loss were included: history of diabetes (none or present) and body mass index (kg/m^2) (≥25.0, 18.5–25.0, or < 18.5). In addition, social determinants variables that could affect oral health were also included: years of education (< 9, 10–12, or > 12 years), self-rated household economic status in early life at 5 years old (rich, fair, or poor), marital status (married or single), and number of family members in the household (living alone, 2, 3, or ≥4).

We supposed potential pathways: income, psychological stress and disorders, access to health care, and health behavior. Annual household income (0–300, 300–750, or > 750 million Japanese yen) was also included. We used feeling fear of job loss (yes or no) and psychological distress (K6 score [19]; none (0–4) or present (≥5)) as a psychological stress and disorders variable. To assess the access to health care, we included visiting a dental clinic for preventative care (yes or no) and hesitation to use medical and dental care (yes, no, or never felt a need to use). We included smoking status (current smoker, former smoker, or never smoker) as a health behavior variable. We created dummy variables for the missing values for each covariate.

### Statistical analysis

**Fig. 2:**
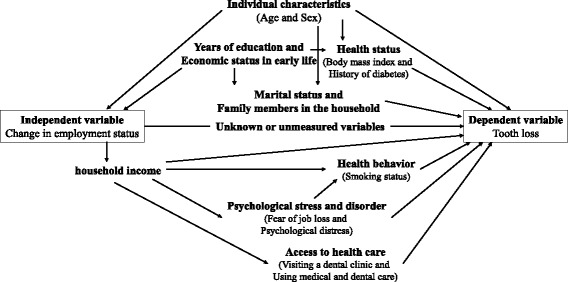
A directed acyclic graph (DAG) showing the association between employment status and tooth loss

We conducted a negative binomial regression analysis stratified by sex to estimate prevalence rate ratios (PRRs) and 95% confidence intervals (95%CIs) for tooth loss, because there are clear different trends of employment status between men and women in Japan [20, 21]. We also examined an interaction term between changes in employment status and sex adjusting for age. We created 2 models for adjusting potential confounders. In model 1, we controlled for age. In model 2, years of education, self-rated household economic status in early life at 5 years old, marital status, and number of family members in the household, history of diabetes, and body mass index were added to model 1. Subsequently, we constructed a model to evaluate how potential pathway variables explain the association. In model 3, we added annual household income to model 2. In model 4, we added visiting a dental clinic for preventive care and hesitation to use medical and dental care to model 3. In model 5, we added feel fear of job loss and psychological distress to model 4. Finally, in model 6, we added smoking status to model 5. We further conducted an analysis using 4 categories of independent variables to validate the findings of the main analysis. In addition, we conducted a linear regression analysis to confirm the validity of the results from a negative binomial regression analysis. We applied a chi-squared test for cross-tabulation. In addition, we constructed a directed acyclic graph (DAG) of proposed associations between employment status and tooth loss to guide our analyses (Fig. 2). *P* values of < 0.05 (two tailed) were considered significant. Analyses were conducted by using STATA ver. 14.2 (Stata Corp., College Station, TX).

## Results

The median age was 37 years (first quartile to third quartile = 31 to 43). More than half of the participants were men (*n* = 1394, 52.6%). The percentage of the experience of temporary employment was 14.5% (*n* = 202) in men and 61.3% (*n* = 771) in women. Tables 1 and 2 show the characteristics and dependent variables among men and women. There was no significant association between men who experienced temporary employment and tooth loss. On the contrary, compared with women who were continuous regular employees, there was a significant association between women who experienced temporary employment and tooth loss.

**Table 1:** Characteristics and tooth loss in men (*n* = 1,394)

Men (*n *= 1,394)			Number of tooth loss						
			none	1 tooth	2 teeth	3 teeth	4 teeth	more than 4 teeth	*P*-value*
Changes in employment status	Continuous regular employment	n	736	140	111	60	46	99	0.68
	(*n* = 1,192)	(%)	(61.7)	(11.7)	(9.3)	(5.0)	(3.9)	(8.3)	
	Having the experience of temporary employment	n	122	20	23	9	6	22	
	(*n* = 202)	(%)	(60.4)	(9.9)	(11.4)	(4.5)	(3.0)	(10.9)	
	Regular to temporary employment	n	32	7	10	7	0	9	
	(*n* = 65)	(%)	(49.2)	(10.8)	(15.4)	(10.8)	(0.0)	(13.9)	
	Temporary to regular employment	n	32	5	7	0	2	8	
	(*n* = 54)	(%)	(59.3)	(9.3)	(13.0)	(0.0)	(3.7)	(14.8)	
	Continuous temporary employment	n	58	8	6	2	4	5	
	(*n* = 83)	(%)	(69.9)	(9.6)	(7.2)	(2.4)	(4.8)	(6.0)	
Age (years old)	25–30	n	220	18	15	3	4	6	<0.05
		(%)	(82.7)	(6.8)	(5.6)	(1.1)	(1.5)	(2.3)	
	30–35	n	169	22	15	11	7	16	
		(%)	(70.4)	(9.2)	(6.3)	(4.6)	(2.9)	(6.7)	
	35–40	n	195	41	27	15	12	19	
		(%)	(63.1)	(13.3)	(8.7)	(4.9)	(3.9)	(6.2)	
	40–45	n	159	45	43	18	17	33	
		(%)	(50.5)	(14.3)	(13.7)	(5.7)	(5.4)	(10.5)	
	45–50	n	115	34	34	22	12	47	
		(%)	(43.6)	(12.9)	(12.9)	(8.3)	(4.6)	(17.8)	
History of diabetes	None	n	843	157	132	67	51	116	0.62
		(%)	(61.7)	(11.5)	(9.7)	(4.9)	(3.7)	(8.5)	
	Present	n	15	3	2	2	1	5	
		(%)	(53.6)	(10.7)	(7.1)	(7.1)	(3.6)	(17.9)	
Body mass index (kg/m^2)	≥25.0	n	214	53	31	20	14	40	0.11
		(%)	(57.5)	(14.3)	(8.3)	(5.4)	(3.8)	(10.8)	
	18.5–25.0	n	601	104	100	44	35	76	
		(%)	(62.6)	(10.8)	(10.4)	(4.6)	(3.7)	(7.9)	
	<18.5	n	38	3	2	5	3	3	
		(%)	(70.4)	(5.6)	(3.7)	(9.3)	(5.6)	(5.6)	
Marital status	Married	n	575	113	99	53	43	93	<0.05
		(%)	(58.9)	(11.6)	(10.1)	(5.4)	(4.4)	(9.5)	
	Single	n	283	47	35	16	9	28	
		(%)	(67.7)	(11.2)	(8.4)	(3.8)	(2.2)	(6.7)	
No. of family members in the household	Living alone	n	109	21	18	13	3	19	0.56
		(%)	(59.6)	(11.5)	(9.8)	(7.1)	(1.6)	(10.4)	
	2	n	163	25	25	10	9	24	
		(%)	(63.7)	(9.8)	(9.8)	(3.9)	(3.5)	(9.4)	
	3	n	228	40	32	14	9	27	
		(%)	(65.1)	(11.4)	(9.1)	(4.0)	(2.6)	(7.7)	
	≥4	n	357	74	59	32	31	51	
		(%)	(59.1)	(12.3)	(9.8)	(5.3)	(5.1)	(8.4)	
Self-rated household economic status in early life at 5 years old	Rich	n	138	45	28	13	13	28	0.06
		(%)	(52.1)	(17.0)	(10.6)	(4.9)	(4.9)	(10.6)	
	Fair	n	566	90	80	45	29	69	
		(%)	(64.4)	(10.2)	(9.1)	(5.1)	(3.3)	(7.9)	
	Poor	n	145	24	25	11	10	23	
		(%)	(60.9)	(10.1)	(10.5)	(4.6)	(4.2)	(9.7)	
Years of education (year)	<9	n	31	3	6	6	1	6	<0.05
		(%)	(58.5)	(5.7)	(11.3)	(11.3)	(1.9)	(11.3)	
	9–12	n	113	32	31	12	8	35	
		(%)	(48.9)	(13.9)	(13.4)	(5.2)	(3.5)	(15.2)	
	>12	n	708	124	96	51	42	80	
		(%)	(64.3)	(11.3)	(8.7)	(4.6)	(3.8)	(7.3)	
Annual household income (million yen)	0–300	n	35	10	7	5	1	9	0.86
		(%)	(52.2)	(14.9)	(10.5)	(7.5)	(1.5)	(13.4)	
	300–750	n	369	67	60	28	23	51	
		(%)	(61.7)	(11.2)	(10.0)	(4.7)	(3.9)	(8.5)	
	≥750	n	287	59	47	26	16	46	
		(%)	(59.7)	(12.3)	(9.8)	(5.4)	(3.3)	(9.6)	
Feel fear of job loss	No	n	563	103	81	43	25	68	<0.05
		(%)	(63.8)	(11.7)	(9.2)	(4.9)	(2.8)	(7.7)	
	Yes	n	269	55	47	23	25	52	
		(%)	(57.1)	(11.7)	(10.0)	(4.9)	(5.3)	(11.0)	
Psychological distress (k6)	None (0-4)	n	565	106	89	45	32	78	0.99
		(%)	(61.8)	(11.6)	(9.7)	(4.9)	(3.5)	(8.5)	
	Present (≥5)	n	293	54	44	24	20	42	
		(%)	(61.4)	(11.3)	(9.2)	(5.0)	(4.2)	(8.8)	
Visiting a dental clinic for preventive care	Yes	n	201	38	24	25	11	27	0.12
		(%)	(61.7)	(11.7)	(7.4)	(7.7)	(3.4)	(8.3)	
	No	n	654	122	109	44	41	93	
		(%)	(61.5)	(11.5)	(10.3)	(4.1)	(3.9)	(8.8)	
Hesitation to use medical and dental care	Yes	n	374	78	58	28	28	69	<0.05
		(%)	(58.9)	(12.3)	(9.1)	(4.4)	(4.4)	(10.9)	
	No	n	353	70	55	34	18	38	
		(%)	(62.2)	(12.3)	(9.7)	(6.0)	(3.2)	(6.7)	
	Never felt a need to use	n	131	12	21	6	6	14	<0.05
		(%)	(69.0)	(6.3)	(11.1)	(3.2)	(3.2)	(7.4)	
Smoking status	Current smoker	n	258	61	62	29	26	58	
		(%)	(52.2)	(12.4)	(12.6)	(5.9)	(5.3)	(11.7)	
	Former smoker	n	226	43	41	22	15	30	
		(%)	(60.0)	(11.4)	(10.9)	(5.8)	(4.0)	(8.0)	
	Never smoker	n	373	56	31	18	11	32	
		(%)	(71.6)	(10.8)	(6.0)	(3.5)	(2.1)	(6.1)	

* *P*-value was calculated by chi-squared test

**Table 2:** Characteristics and tooth loss in women (*n* = 1,258)

Women (*n* =1,258)			Number of tooth loss						
			none	1 tooth	2 teeth	3 teeth	4 teeth	more than 4 teeth	*P*-value*
Changes in employment status	Continuous regular employment	n	349	63	29	15	12	19	<0.05
	(*n* = 487)	(%)	(71.7)	(12.9)	(6.0)	(3.1)	(2.5)	(3.9)	
	Having the experience of temporary employment	n	449	116	66	47	26	67	
	(*n* = 771)	(%)	(58.2)	(15.1)	(8.6)	(6.1)	(3.4)	(8.7)	
	Regular to temporary employment	n	286	82	52	33	17	47	
	(*n* = 517)	(%)	(55.3)	(15.9)	(10.1)	(6.4)	(3.3)	(9.1)	
	Temporary to regular employment	n	39	7	2	2	0	5	
	(*n* = 55)	(%)	(70.9)	(12.7)	(3.6)	(3.6)	(0.0)	(9.1)	
	Continuous temporary employment	n	124	27	12	12	9	15	
	(*n* = 199)	(%)	(62.3)	(13.6)	(6.0)	(6.0)	(4.5)	(7.5)	
Age (years old)	25–30	n	248	20	8	5	6	5	<0.05
		(%)	(84.9)	(6.9)	(2.7)	(1.7)	(2.1)	(1.7)	
	30–35	n	163	24	12	6	7	9	
		(%)	(73.8)	(10.9)	(5.4)	(2.7)	(3.2)	(4.1)	
	35–40	n	152	47	21	8	4	16	
		(%)	(61.3)	(19.0)	(8.5)	(3.2)	(1.6)	(6.5)	
	40–45	n	133	47	25	16	11	19	
		(%)	(53.0)	(18.7)	(10.0)	(6.4)	(4.4)	(7.6)	
	45–50	n	102	41	29	27	10	37	
		(%)	(41.5)	(16.7)	(11.8)	(11.0)	(4.1)	(15.0)	
History of diabetes	None	n	793	178	94	60	38	86	0.24
		(%)	(63.5)	(14.3)	(7.5)	(4.8)	(3.0)	(6.9)	
	Present	n	5	1	1	2	0	0	
		(%)	(55.6)	(11.1)	(11.1)	(22.2)	(0.0)	(0.0)	
Body mass index (kg/m^2)	≥25.0	n	65	19	14	7	4	18	<0.05
		(%)	(51.2)	(15.0)	(11.0)	(5.5)	(3.2)	(14.2)	
	18.5–25.0	n	580	131	63	43	27	60	
		(%)	(64.2)	(14.5)	(7.0)	(4.8)	(3.0)	(6.6)	
	<18.5	n	113	25	13	11	7	7	
		(%)	(64.2)	(14.2)	(7.4)	(6.3)	(4.0)	(4.0)	
Marital status	Married	n	455	124	58	51	27	62	<0.05
		(%)	(58.6)	(16.0)	(7.5)	(6.6)	(3.5)	(8.0)	
	Single	n	340	55	37	11	11	24	
		(%)	(71.1)	(11.5)	(7.7)	(2.3)	(2.3)	(5.0)	
No. of family members in the household	Living alone	n	75	6	7	5	3	5	0.32
		(%)	(74.3)	(5.9)	(6.9)	(5.0)	(3.0)	(5.0)	
	2	n	161	43	15	10	6	21	
		(%)	(62.9)	(16.8)	(5.9)	(3.9)	(2.3)	(8.2)	
	3	n	201	42	34	15	12	22	
		(%)	(61.7)	(12.9)	(10.4)	(4.6)	(3.7)	(6.8)	
	≥4	n	356	87	39	31	17	37	
		(%)	(62.8)	(15.3)	(6.9)	(5.5)	(3.0)	(6.5)	
Self-rated household economic status in early life at 5 years old	Rich	n	139	32	26	18	12	28	<0.05
		(%)	(54.5)	(12.6)	(10.2)	(7.1)	(4.7)	(11.0)	
	Fair	n	490	111	57	33	16	42	
		(%)	(65.4)	(14.8)	(7.6)	(4.4)	(2.1)	(5.6)	
	Poor	n	162	35	12	11	10	15	
		(%)	(66.1)	(14.3)	(4.9)	(4.5)	(4.1)	(6.1)	
Years of education (year)	<9	n	17	6	1	6	2	3	<0.05
		(%)	(48.6)	(17.1)	(2.9)	(17.1)	(5.7)	(8.6)	
	9–12	n	127	37	25	15	11	25	
		(%)	(52.9)	(15.4)	(10.4)	(6.3)	(4.6)	(10.4)	
	>12	n	647	135	68	41	25	57	
		(%)	(66.5)	(13.9)	(7.0)	(4.2)	(2.6)	(5.9)	
Annual household income (million yen)	0–300	n	53	9	5	3	3	12	0.41
		(%)	(62.4)	(10.6)	(5.9)	(3.5)	(3.5)	(14.1)	
	300–750	n	249	58	39	21	9	28	
		(%)	(61.6)	(14.4)	(9.7)	(5.2)	(2.2)	(6.9)	
	≥750	n	233	57	26	20	14	27	
		(%)	(61.8)	(15.1)	(6.9)	(5.3)	(3.7)	(7.2)	
Feel fear of job loss	No	n	495	123	56	38	23	45	0.09
		(%)	(63.5)	(15.8)	(7.2)	(4.9)	(3.0)	(5.8)	
	Yes	n	272	46	32	21	13	39	
		(%)	(64.3)	(10.9)	(7.6)	(5.0)	(3.1)	(9.2)	
Psychological distress (k6)	None (0-4)	n	548	120	66	30	25	50	<0.05
		(%)	(65.3)	(14.3)	(7.9)	(3.6)	(3.0)	(6.0)	
	Present (≥5)	n	248	59	29	31	13	36	
		(%)	(59.6)	(14.2)	(7.0)	(7.5)	(3.1)	(8.7)	
Visiting a dental clinic for preventive care	Yes	n	247	57	40	27	18	20	<0.05
		(%)	(60.4)	(13.9)	(9.8)	(6.6)	(4.4)	(4.9)	
	No	n	548	122	55	35	20	65	
		(%)	(64.9)	(14.4)	(6.5)	(4.1)	(2.4)	(7.7)	
Hesitation to use medical and dental care	Yes	n	366	80	43	34	13	39	0.45
		(%)	(63.7)	(13.9)	(7.5)	(5.9)	(2.3)	(6.8)	
	No	n	310	77	36	23	17	39	
		(%)	(61.8)	(15.3)	(7.2)	(4.6)	(3.4)	(7.8)	
	Never felt a need to use	n	122	22	16	5	8	8	
		(%)	(67.4)	(12.2)	(8.8)	(2.8)	(4.4)	(4.4)	
Smoking status	Current smoker	n	91	26	15	17	11	21	<0.05
		(%)	(50.3)	(14.4)	(8.3)	(9.4)	(6.1)	(11.6)	
	Former smoker	n	115	34	20	12	5	20	
		(%)	(55.8)	(16.5)	(9.7)	(5.8)	(2.4)	(9.7)	
	Never smoker	n	589	119	59	33	22	45	
		(%)	(67.9)	(13.7)	(6.8)	(3.8)	(2.5)	(5.2)	

* *P*-value was calculated by chi-squared test

Table 3 shows the associations between change in employment status and tooth loss found with the multivariable ordered logistic regression models stratified by sex. We found no significant interaction between employment status and sex after adjusting for age (*p* = 0.71). In model 1, we confirmed a significant association between the experience of temporary employment and tooth loss in both sexes. Model 2 also showed that the experience of temporary employment was significantly associated with tooth loss after adjusting for potential confounders (men: PRR = 1.50 [95%CI = 1.13, 2.00]; women: PRR = 1.42 [95%CI = 1.14, 1.76]). In the additional analysis, compared with continuous regular employment, changes from regular to temporary employment and temporary to regular employment as well as continuous temporary employment were associated with tooth loss in models 1 and 2.

**Table 3:** Associations between change in employment status and tooth loss

	Changes in employment status				
	Continuous regular employment	Having the experience of temporary employment	Regular to temporary employment	Temporary to regular employment	Continuous temporary employment
Negative binomial regression models	Reference	PRR (95%CI)	PRR (95%CI)	PRR (95%CI)	PRR (95%CI)
Men (*n*=1,394)	(*n*=1,192)	(*n*=202)	(*n*=65)	(*n*=54)	(*n*=83)
Model 1	1.00	1.55 (1.18, 2.04)	1.71 (1.11, 2.63)	1.69 (1.05, 2.73)	1.31 (0.86, 2.01)
Model 2	1.00	1.50 (1.13, 2.00)	1.62 (1.05, 2.52)	1.62 (0.99, 2.64)	1.30 (0.83, 2.02)
Model 3	1.00	1.44 (1.07, 1.93)	1.51 (0.96, 2.37)	1.63 (1.00, 2.65)	1.22 (0.77, 1.92)
Model 4	1.00	1.38 (1.03, 1.85)	1.44 (0.91, 2.26)	1.53 (0.94, 2.50)	1.20 (0.76, 1.88)
Model 5	1.00	1.32 (0.98, 1.78)	1.37 (0.87, 2.16)	1.46 (0.89, 2.39)	1.16 (0.74, 1.82)
Model 6	1.00	1.31 (0.98, 1.76)	1.41 (0.90, 2.21)	1.43 (0.88, 2.33)	1.13 (0.72, 1.77)
Women (n=1,258)	(*n*=487)	(*n*=771)	(*n*=517)	(*n*=55)	(*n*=199)
Model 1	1.00	1.44 (1.16, 1.79)	1.34 (1.06, 1.70)	1.33 (0.79, 2.24)	1.73 (1.28, 2.34)
Model 2	1.00	1.42 (1.14, 1.76)	1.35 (1.07, 1.72)	1.30 (0.77, 2.18)	1.62 (1.19, 2.19)
Model 3	1.00	1.37 (1.10, 1.71)	1.31 (1.02, 1.66)	1.31 (0.78, 2.20)	1.56 (1.14, 2.12)
Model 4	1.00	1.38 (1.11, 1.72)	1.32 (1.03, 1.68)	1.29 (0.76, 2.19)	1.58 (1.16, 2.15)
Model 5	1.00	1.37 (1.09, 1.71)	1.32 (1.03, 1.70)	1.27 (0.75, 2.17)	1.51 (1.10, 2.06)
Model 6	1.00	1.33 (1.06, 1.66)	1.31 (1.02, 1.68)	1.14 (0.67, 1.94)	1.44 (1.06, 1.97)
Linear regression models	Reference	Coefficient (95%CI)	Coefficient (95%CI)	Coefficient (95%CI)	Coefficient (95%CI)
Men (*n*=1,394)	(*n*=1,192)	(*n*=202)	(*n*=65)	(*n*=54)	(*n*=83)
Model 1	-	0.38 (0.14, 0.62)	0.51 (0.12, 0.91)	0.47 (0.04, 0.90)	0.21 (-0.15, 0.57)
Model 2	-	0.37 (0.12, 0.62)	0.46 (0.06, 0.85)	0.42 (-0.01, 0.85)	0.25 (-0.12, 0.62)
Model 3	-	0.34 (0.09, 0.59)	0.41 (0.01, 0.81)	0.42 (-0.02, 0.85)	0.22 (-0.16, 0.59)
Model 4	-	0.32 (0.07, 0.57)	0.38 (-0.02, 0.78)	0.38 (-0.05, 0.81)	0.23 (-0.15, 0.60)
Model 5	-	0.28 (0.02, 0.53)	0.33 (-0.07, 0.74)	0.31 (-0.12, 0.74)	0.20 (-0.18, 0.58)
Model 6	-	0.25 (0.00, 0.50)	0.31 (-0.09, 0.71)	0.29 (-0.15, 0.72)	0.17 (-0.20, 0.55)
Women (*n*=1,258)	(*n*=487)	(*n*=771)	(*n*=517)	(*n*=55)	(*n*=199)
Model 1	-	0.25 (0.08, 0.41)	0.19 (0.00, 0.38)	0.25 (-0.16, 0.65)	0.36 (0.13, 0.60)
Model 2	-	0.23 (0.06, 0.40)	0.20 (0.00, 0.39)	0.25 (-0.15, 0.65)	0.31 (0.07, 0.54)
Model 3	-	0.20 (0.03, 0.38)	0.17 (-0.03, 0.36)	0.24 (-0.16, 0.64)	0.27 (0.02, 0.51)
Model 4	-	0.21 (0.04, 0.39)	0.17 (-0.02, 0.37)	0.24 (-0.16, 0.65)	0.28 (0.04, 0.53)
Model 5	-	0.20 (0.02, 0.38)	0.16 (-0.04, 0.36)	0.25 (-0.15, 0.65)	0.26 (0.01, 0.51)
Model 6	-	0.16 (-0.02, 0.33)	0.13 (-0.06, 0.33)	0.11 (-0.29, 0.51)	0.22 (-0.03, 0.46)

Model 1: Age was adjusted

Model 2: Model 1 + years of education, self-rated household economic status in early life at 5 years old, marital status, no. of family members in the household, history of diabetes, and body mass index were adjusted

Model 3: Model 2 + Annual household income was adjusted

Model 4: Model 3 + Visiting a dental clinic for preventive care and hesitation to use medical and dental care were adjusted

Model 5: Model 4 + Feel fear of job loss and psychological distress was adjusted

Model 6: Model 5 + Smoking status was adjusted

*Abbreviation*: *PRR* prevalence rate ratios, *95%CI* 95% confidence interval

In models 3 to 6, we observed associations between changes in employment status and tooth loss after adjusting for potential pathway variables. Compared with continuous regular employment, the PRR of having the experience of temporary employment decreased in models 3 to 6 (men, PRR = 1.44 [95%CI = 1.07, 1.93] to 1.31 [95%CI = 0.98, 1.76]; women, PRR = 1.37 [95%CI = 1.10, 1.71] to 1.33 [95%CI = 1.06, 1.66]). Similar trends were observed in the additional analysis of the regular to temporary employment, temporary to regular employment, and continuous temporary employment groups. The results from the linear regression analysis also showed similar trends with the main analysis.

## Discussions

The results of our study showed that the experience of temporary employment was associated with tooth loss in both men and women in Japan. In addition, changes from regular to temporary employment and temporary to regular employment as well as continuous temporary employment were associated with tooth loss.

The association between temporary employment and poor oral health is important in public health because the level of unstable employment is increasing in both the private and public sectors in many developed countries [5]. The number of temporary employees continues to increase in these countries [9]: for example, the proportion of temporary employees in Japan was only 18.3% in 1988 but reached 37.4%, or more than 1 in 3 workers, in 2014 [22]. Furthermore, more than half of employed young people (15–24 years old) in certain European countries are temporary workers: 53.6% in Germany, 57.1% in Italy, and 59.6% in France in 2015 [23]. Dental health professionals and public policy makers should understand the enormous impact of increasing temporary employment on tooth loss.

We found that temporary employment was associated with tooth loss among both male and female workers in Japan. A previous survey of the labor force showed that the reasons for being temporarily employed differ between men and women. The primary reasons for temporary employment in men were “Can’t find regular employment jobs” (26.9%), whereas the reason in women was “work only during convenient time” (27.6%) [24]. Therefore, it is conceivable that the association between temporary employment and oral health would also differ between sexes. That is, the negative effect of being temporarily employed would be amplified in men. However, the evidence suggests a different effect. Inoue et al. reported that temporary female employees faced precarious situations such as low income, limited social safety net, and difficulty sustaining work–life balance [21]. The current study also revealed that female participants who experienced temporary employment were low paid and fearful about job loss. Therefore, temporary employment could affect tooth loss in both sexes uniformly.

Several potential pathways can exist between temporary employment and oral health. First, economic factors may link employment status and oral health. In general, temporary employees have incomes lower than those of regular employees, and low income is among the key risk factors for oral disease [25]. Low income is associated with severe caries and periodontal disease, and poor people are less likely to use medical services [26]. Indeed, the association between temporary employment and tooth loss was explained by the analysis of income in the present study (models 2 and 3).

Second, psychological stress and disorders may explain the association between temporary employment and tooth loss. Because they can be easily dismissed, temporary employees tend to feel more job insecurity and work-related stress which lead to psychological disorders [7, 13, 27]. Stress from fear of job loss and psychological disorders could influence health behaviors such as less frequent toothbrushing and heavier smoking [11]. In addition, stress may decrease salivary flow, which increases the occurrence and progression of periodontal disease [12]. Temporary employees could lose their teeth for any of these reasons. Indeed, the association between temporary employment and tooth loss was explained by the fear of job loss and psychological disorders in the present analysis (models 4 and 5).

Third, poor health behavior also might explain the association between employment status and oral health. Work stress was associated with poor health behaviors such as less frequent toothbrushing and heavy smoking [11]. In addition, low social economic status could lead to poor oral health behaviors [26]. Indeed, the association between temporary employment and tooth loss was explained by smoking status (models 5 and 6). However, we could not obtain data on oral health behavior variables such as toothbrushing. It might also well explain the association between temporary employment and tooth loss.

Finally, limited access to health care might explain the association between employment status and oral health. Japan has universal healthcare coverage (UHC) and patients pay only 10–30% of the total cost of treatment [28]. Also, the total cost itself is relatively low because the cost is controlled by the government. In addition, the UHC covers the most basic dental treatments, such as treatments for caries and periodontal disease [28]. With the UHC, most people in Japan did not hesitate obtaining medical and dental services. However, under long lasting economic depression, some people in temporary employment, a new emerging type of unstable employment, were not able to use health care service appropriately due to the following two reasons [29]; 1) even 10–30% of the total cost of dental care could be a barrier for them to use dental care because they were employed at a low wage, 2) they may be reluctant to take a time off from work to visit dental services because they are concerned that they might be fired if they are absent frequently owing to sickness. Indeed, the association between temporary employment and tooth loss was explained by the frequency of visiting a dental clinic for preventive care and the hesitation to use medical and dental care as analyzed in our study (models 3 and 4).

The present study has limitations. First, both the independent and dependent variables were self-reported, which may have introduced self-reporting bias. Although, several studies have shown that the validity and reliability of self-reported oral health status are acceptable [30], self-rated number of teeth lost is not validated. However, previous studies have used self-reported number of teeth lost [31, 32]. Second, the response rate was relatively low, which could be another source of bias. However, the respondents had characteristics that were fairly comparable to those of the target population [18]. Therefore, our findings are likely to be generalizable in Japan.

## Conclusions

In conclusion, we found a significant association between temporary employment and tooth loss. A previous study indicated that there is a need to enhance the social safety net for temporary employees even in high-income countries [5]. Secure employment is a social determinant of health [5], and the assurance of safety/physical protections in workplaces, health insurance, and more stable employment arrangements are needed. Policy makers as well as dental health professionals should understand the impact of employment status on population health.

## Abbreviations

95%CI: 95% confidence interval
J-SHINE: Japanese Study on Stratification, Health, Income, and Neighborhood
PRR: Prevalence rate ratios
UHC: universal healthcare coverage
WHO: World Health Organization
